# Different AHO phenotype in a Chinese family with a novel *GNAS* missense variant: a case report

**DOI:** 10.1186/s13052-022-01322-6

**Published:** 2022-07-23

**Authors:** Qing Zhou, Bin Liang, Qing-Xian Fu, Hui Liu, Chao-Chun Zou

**Affiliations:** 1 Department of Endocrinology and Inborn Metabolic Diseases, Fujian Maternity and Child Health Hospital, Fujian Province, Fuzhou, 350000 China; 2 Medical Genetic Diagnosis and Therapy Center, Fujian Key Laboratory for Prenatal Diagnosis and Birth Defect, Fujian Maternity and Child Health Hospital, Fuzhou, China; 3 Department of Endocrinology and Inborn Metabolic Diseases, Fujian Children’s Hospital, College of Clinical Medicine for Obstetrics & Gynecology and Pediatrics, Fujian Medical University, Fuzhou, China; 4 Department of Endocrinology, Children’s Hospital of Zhejiang University School of Medicine, Zhejiang Province, Hangzhou, 310000 China

**Keywords:** Pseudohypoparathyroidism, Pseudo-pseudohypoparathyroidism, *GNAS* gene, Albright hereditary osteodystrophy

## Abstract

**Background:**

Albright’s hereditary osteodystrophy (AHO) is an inherited disorder which is caused by an inactivating variant in the *GNAS* gene. AHO appears associated to either pseudohypoparathyroidism 1a (PHP1a) when *GNAS* gene is maternally inherited or to pseudo-pseudohypoparathyroidism (PPHP) when it is paternally inherited. We describe the clinical and biochemical characteristics of two patients, a boy and his mother with a novel heterozygous missense variant of *GNAS* gene.

**Case presentation:**

The boy presented with typical AHO phenotype (early-onset obesity, round face, short neck, shortened fifth metacarpal bone, developmental retardation, but without short stature and subcutaneous calcifications), multiple hormone resistance including PTH, TSH and ACTH, and mild calcification in the right basal ganglia. The mother only presented with brachydactyly and short stature, without hormone resistance and other signs of AHO. Whole-exome sequencing identified in the son and his mother a novel heterozygous missense variant (p. Val375Leu) in exon 13 of *GNAS* gene. The diagnosis of PHP-1a for the son and PPHP for the mother were confirmed.

**Conclusion:**

This study further expands the spectrum of known *GNAS* pathogenic variants, and also demonstrates the heterogeneous phenotype of AHO due to a novel *GNAS* pathogenic variant.

## Introduction

Albright’s hereditary osteodystrophy (AHO) is an autosomal dominant syndrome characterized by obesity, short stature, round face, brachydactyly, heterotopic ossifications, and cognitive impairment, caused by loss-of-function pathogenic variants in *GNAS* gene [1]. *GNAS* gene encodes the stimulatory G-protein α-subunit (G_s_α), which is expressed in nearly all cells. But in some specific hormone target tissues such as proximal renal tubules, pituitary, thyroid, and gonads, G_s_α is expressed primarily from the maternal allele [2]. G_s_α couples many receptors for hormones and catalyzes the conversion of ATP into the second messenger cAMP. Therefore, inactivating *GNAS* pathogenic variants from maternal allele leads to a dramatic reduction in G_s_α expression, characterized by end-organ resistance to multiple hormones, primarily PTH and TSH, causing hypocalcemia, hyperphosphatemia, elevated PTH levels and hypothyroidism [3].

AHO accompanied with end-organ resistance to hormone is termed pseudohypoparathyroidism type 1a (PHP1a), whereas AHO with a normal biochemical profile is known as pseudo-pseudohypoparathyroidism (PPHP) [4]. AHO phenotype is caused by haplotype insufficiency of *GNAS* gene in patients either with PHP1a or PPHP [5]. PHP1a is the most common variant, caused by loss-of-function pathogenic variant in maternally inherited *GNAS* gene, on the contrary, PPHP is caused by loss-of-function pathogenic variant in paternally inherited *GNAS* gene [6].

Here we report a 9-month-old male and his mother who both share a novel pathogenic variant in exon 13 of the *GNAS* gene. The diagnosis of PHP1a for the son and PPHP for the mother were confirmed. However, their AHO phenotypes were quite different.

## Case presentation

The proband was born at 33^+4^ weeks with a weight of 1.8 kg. At the age of 21 days, with a normal thyroid indicated by Doppler ultrasound, he was diagnosed with primary hypothyroidism (Table 1), and treated with low dose levothyroxine (3 μg/kg). At the age of 8 months, he was hospitalized due to septic shock and severe pneumonia for more than 3 weeks in the local hospital. Family history revealed that his mother had short stature (150 cm, P3) and that his father, sister and grandparents were all normal.

**Table 1:** Laboratory evaluations of the proband

Parameters	Normal range	Chronological age	
		21 days	9 months
Calcium	2.25 ~ 2.75 mmol/L	/	1.72
Phosphate	1.25 ~ 2.10 mmol/L	/	1.35
PTH	1.6 ~ 6.9 pmol/L	/	11.7
FT4	12 ~ 22 pmol/L	9.88	13.32
FT3	3.1 ~ 6.8 pmol/L	3.79	2.34
TSH	0.27 ~ 4.2 mIU/L	10.27	6.19
ACTH (8 am)	1.6 ~ 13.9 pmol/L	/	34.97
Cortisol (8 am)	138 ~ 635 nmol/L	/	500.9
Cortisol (4 pm)	82 ~ 413 nmol/L	/	138.7

*PTH* Parathyroid hormone, *FT3* Free triiodothyronine, *FT4* Free thyroxine,

*TSH* Thyroid-stimulating hormone, *ACTH* Adrenocorticotropic hormone

**Fig. 1:**
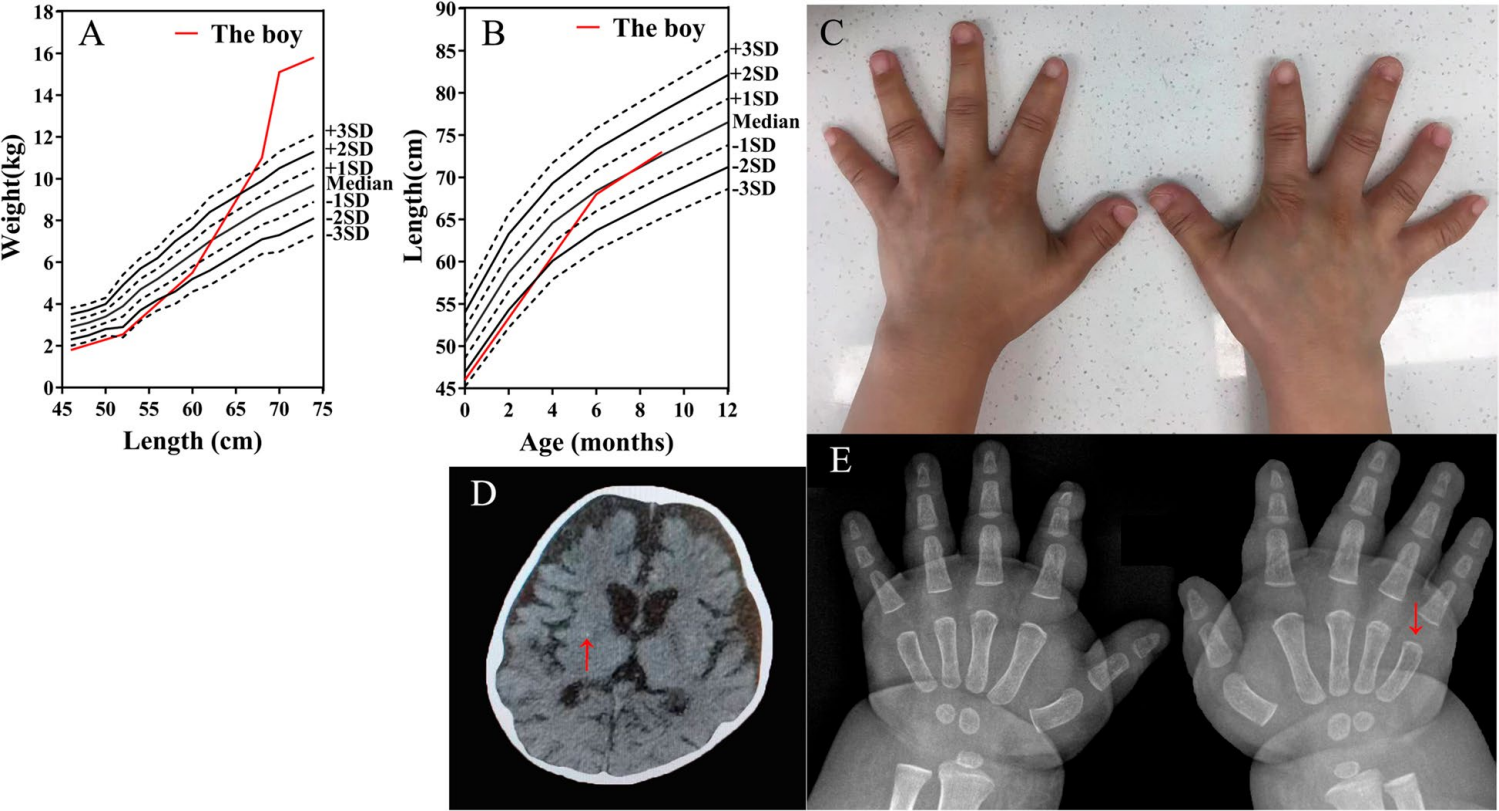
Clinical phenotype of the proband and his mother. **A** Weight-for-length Z-score curve of the proband according to the standards for children in China. The red color represents the proband’s accelerated weight gain. **B** Length-for-age Z-score curve of the proband according to the standards for children in China. **C** Brachydactyly from the proband’s mother. **D** Cranial computed tomography of the proband demonstrated right focal basal ganglia calcification (arrow). **E** Radiograph of the hands demonstrated the proband’s shortened 5th metacarpal on the right hand (arrow)

On physical examination at 9 months of age, his body weight was 15.6 kg (> 3 SD) and height 73 cm (about P50). The proband’s weight-for-length Z-score curve is shown in Fig. 1A, and length-for-age Z-score curve is shown in Fig. 1B. He had round face, short neck, brachydactyly and a 3-month delay in motor development. At that time he did not have subcutaneous calcifications nor café-au-lait spots. Physical examination of his mother showed, apart from her short stature, a brachydactyly (Fig. 1C). She was not dysmorphic nor obese (BMI 21.7 kg/m^2^).

Laboratory tests revealed hypocalcemia, normal serum phosphate, and elevated serum PTH (Table 1). Thyroid function showed normal FT4, low FT3, and elevated TSH (Table 1). He was negative for anti-thyroid peroxidase antibody and thyroglobulin antibody. Elevated ACTH and normal cortisol (Table 1). Plasma renin activity, serum aldosterone level, serum ammonium, serum lactic acid, serum lipid, serum sodium, serum potassium, liver and renal function were all normal. Conventional G-banded karyotype was normal (46, XY). Cranial computed tomography scan discovered mild heterotopic calcification in the right basal ganglia (Fig. 1D). Hand’s X-ray demonstrated shortened fifth metacarpal bone on the right hand (Fig. 1E). Further testing for his mother showed normal serum calcium, phosphate and PTH levels.

**Fig. 2:**
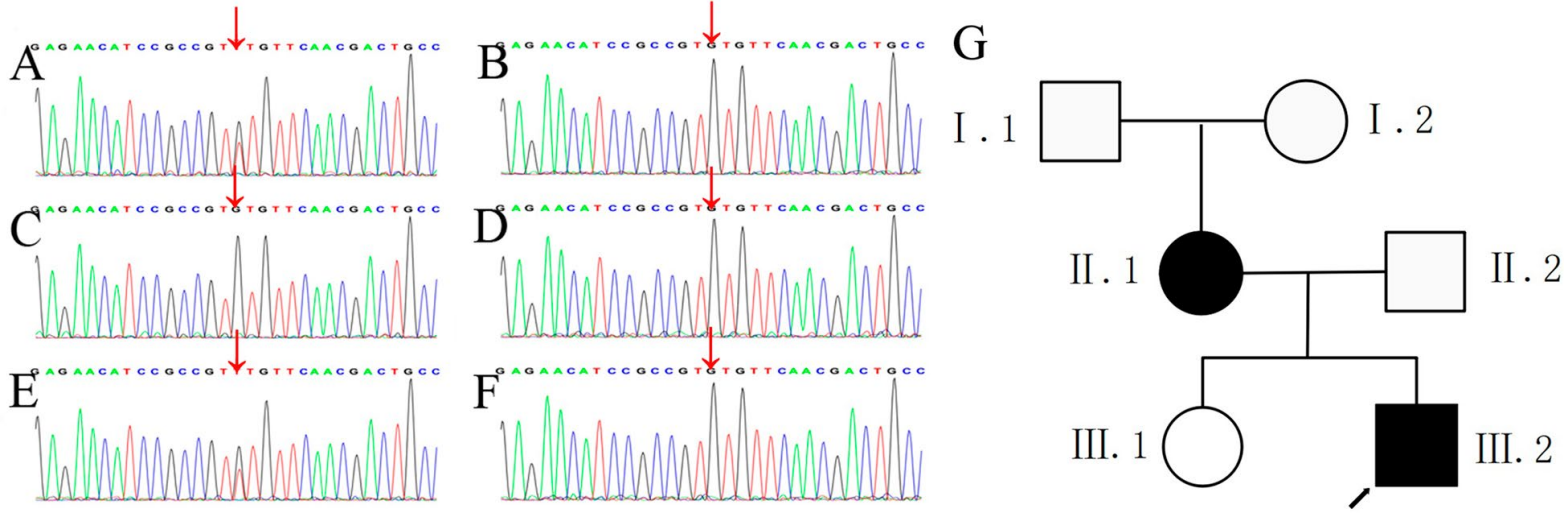
The novel “de novo*”* pathogenic variant in the *GNAS* gene identified in the family. Sequencing analysis of exon 13 shows a heterozygous missense pathogenic variant (c. 1123G > T) (arrow) in the proband (**A**) and his mother (**E**). The variant was absent in his father (**C**), sister (**D**), grandmother (**B**) and grandfather (**F**). Pedigree of the family showing the transmission of the *GNAS* variant (**G**)

The patient was suspected of having a genetic endocrine disorder. Therefore, whole-exome sequencing (WES) was performed to identify the genetic cause. A heterozygous variant in the *GNAS* gene was identified in the proband. It was a missense variant in exon 13 (c.1123G > T) that generates an amino acid conversion (p.Val375Leu), which was not included in the gnomAD and HGMD databases. To the best of our knowledge, the variant has not been previously reported. The identified *GNAS* variant was studied in the family members using Sanger sequencing (Fig. 2A-F). Results revealed that the missense variant was inherited from the mother, but it was absent in the patient’s maternal grandparents, Therefore, it was “de novo” in the patient’s mother. Pedigree of the family is shown in Fig. 2G. After sequencing, functional prediction of the Val375Leu variant demonstrated a harmful effect on the *GNAS* protein resulting from PolyPhen-2 (probably damaging; score, 1), PROVEAN (deleterious; score, -2.87), SIFT (damaging; score, 0.001), Mutation Taster (disease causing; score, 1). According to ACMG standards and guidelines (2015), and the variant was classified as likely pathogenic (PM1 + PM2 + PM6 + PP3).

Based on the typical AHO phenotype, laboratory and radiological findings, the proband was diagnosed with PHP1a. He was then started on calcium and calcitriol replacement. In order to further support the diagnosis of PHP1a, methylation status of *GNAS* gene was assessed by MLPA, and no *GNAS* methylation changes were detected in the proband.

## Discussion and conclusions

PHP1a is a clinical entity mainly caused by maternally transmitted heterozygous inactivating molecular defects of the *GNAS* gene, leading to impaired G protein-coupled receptor (GPCR) signaling [7]. Here, we report a Chinese boy with clinical features of AHO (early-onset obesity, round face, short neck, shortened fifth metacarpal bone, developmental retardation, but without short stature and subcutaneous calcifications), and resistance to PTH, TSH and ACTH. A novel heterozygous missense variant in exon 13 of *GNAS* gene was identified in the proband by WES and Sanger sequencing demonstrated that the proband inherited the variant from his mother, in whom it was “de novo”, since it was not present in her parents.

The distinctive phenotype of PHP-Ia versus PPHP is the result of the G_s_α preferential expression from the maternal allele in specific hormone target tissues, so pathogenic variants on the active maternal allele lead to severe G_s_α deficiency, whereas the same variants on the relatively inactive paternal allele have little effect on G_s_α expression [5]. The proband was diagnosed with PHP1a, whereas his mother, who carried the same mutation, was diagnosed of PPHP, because she had a partial AHO phenotype (short stature and brachydactyly) and no hormone resistance. Therefore, we hypothesized that she carried her de novo *GNAS* variant in her paternal allele.

Cho et al. [8] reported a girl with PHP1a who inherited a heterozygous missense variant in *GNAS* gene from her mother with PPHP, both reported to be obese. Classically, the obesity in AHO for PHP1a and PPHP has been thought to be similar. However, in this report, the proband had accelerated weight gain during the first 7 months of life that led to severe obesity. For the proband’s mother, the clinical features of AHO included short stature but no obesity. Animal studies have shown that when the mutant *GNAS* allele is maternal in origin, obesity is usually present, and often severe, but when the mutant allele is paternal in origin, obesity is not frequent [9]. Clinical investigations also indicate that obesity in the AHO phenotype is significantly more common in PHP1a than in PPHP [5]. The early-onset obesity in PHP1a but not in PPHP could be explained by the evidence that G_s_α is imprinted in the paraventricular nucleus of the hypothalamus [10], since maternal G_s_α variant impaired the G_s_α-coupled melanocortin 4 receptor (MC4R) signaling, *MC4R* pathogenic variants are a common genetic cause of early-onset obesity [7].

Short stature has been considered as one of the most common features of the AHO phenotype in PHP1a. However, in our family, the proband has normal stature, whereas his mother was short. The pathogenetic mechanism of reduced growth in PHP1a was initially thought to be due to GH deficiency caused by GHRH resistance [11]. However, a study showed that in a cohort of ten children with PHP1a and inactivating *GNAS* variants, the height and growth velocity did not significantly differ between GH-deficient and GH-sufficient subjects [12]. The clinical variability of height in children with *GNAS* inactivation variants could be explained by the effect of Gsα on GPCR signaling. Since *GNAS* inactivation variants impair MC4R signaling, even with the suppression of GH release, MC4R deficiency result in accelerated growth [13]. So impaired MC4R signaling may counterbalance the negative effect of partial GH deficiency of growth in early childhood, whereas between 12 and 18 years of age, reduced final height is the result of reduced pubertal growth spurt, which is caused by reduction in Gsα expression impairing the GHRH receptor signaling.

Heterotopic ossifications have been described as part of the phenotype of AHO [6], presenting in patients with either PHP1a or PPHP. It is known that the prevalence of subcutaneous ossifications is approximately the same in both PHP1a and PPHP [14]. Due to the same heterozygous insertion variant in *GNAS* gene, both mother with PPHP and her daughter with PHP1a presented with cerebral calcifications [15]. Whereas the proband in this study presented with mild intracerebral calcifications, and his mother had not heterotopic ossifications. The intrafamilial difference in heterotopic ossifications with the same *GNAS* gene pathogenic variant add further evidence to the variable AHO.

To the best of our knowledge, no cases with different AHO phenotypes between PHP1a and PPHP caused by the same *GNAS* pathogenic variant have been reported among members of the same family. In summary, we report a Chinese boy infant with PHP1a and his mother with PPHP, in whom a novel missense *GNAS* pathogenic variant was identified, but their AHO phenotypes were quite different. The variant in this case has not been reported in the literature, and further expands the heterogeneous phenotype of AHO due to a novel *GNAS* pathogenic variant.

## Data Availability

Not applicable.

## Abbreviations

PHP: Pseudohypoparathyroidism
PPHP: Pseudo PHP
AHO: Albright’s hereditary osteodystrophy
Gsα: Stimulatory G-protein α-subunit
WES: Whole-exome sequencing
GPCR: G protein-coupled receptor
MC4R: Melanocortin 4 receptor
